# Crystal structure of the α-racemate of methohexital

**DOI:** 10.1107/S205698901500105X

**Published:** 2015-01-24

**Authors:** Thomas Gelbrich, Ulrich J. Griesser

**Affiliations:** a Institute of Pharmacy, University of Innsbruck, Innrain 52c, 6020 Innsbruck, Austria

**Keywords:** crystal structure, barbiturate, hydrogen bonding, anaesthetic

## Abstract

Mol­ecules of the title compound, C_14_H_18_N_2_O_3_ [systematic name: 5-allyl-5-(hex-3-yn-2-yl)-1-methylpyrimidine-2,4,6(1*H*,3*H*,5*H*)-trione in the (*R_b_S_h_*)/(*S_b_R_h_*) racemic form], are connected by mutual N—H⋯O=C hydrogen bonds in which the carbonyl group at the 2-position of the pyrimidine­trione ring is employed. These inter­actions result in an inversion dimer which displays a central *R*_2_^2^(8) ring motif. This dimer is topologically distinct from that of the previously reported (*S_b_R_h_*) form, which is, however, also based on an *R*_2_^2^(8) motif. The methyl group at the 1-position of the pyrimidine­trione ring in the title structure is disordered over two sets of sites in a 0.57 (2):0.43 (2) ratio.

## Abstract 2

N—H⋯O=C bonded mol­ecules of the title compound are linked into a inversion dimer with an 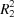
(8) motif.

## Chemical context   

The title compound is a barbiturate derivative, the Na salt of which (trade name Brevimytal, Eli Lilly) is a widely used short-acting anaesthetic with a rapid onset of action. The mol­ecule contains two asymmetric centres and can exist as two diastereomeric enanti­omer pairs. Its stereoisomerism is known to affect the anaesthetic activity and possible side effects of the drug (Gibson *et al.*, 1959 ▸). The crystal structure of the (*S_b_R_h_*) form of methohexital was previously reported by Brunner *et al.* (2003 ▸), who also established that the commercial product (α-racemate) consists of the (*R_b_S_h_*) and (*S_b_R_h_*) isomers.
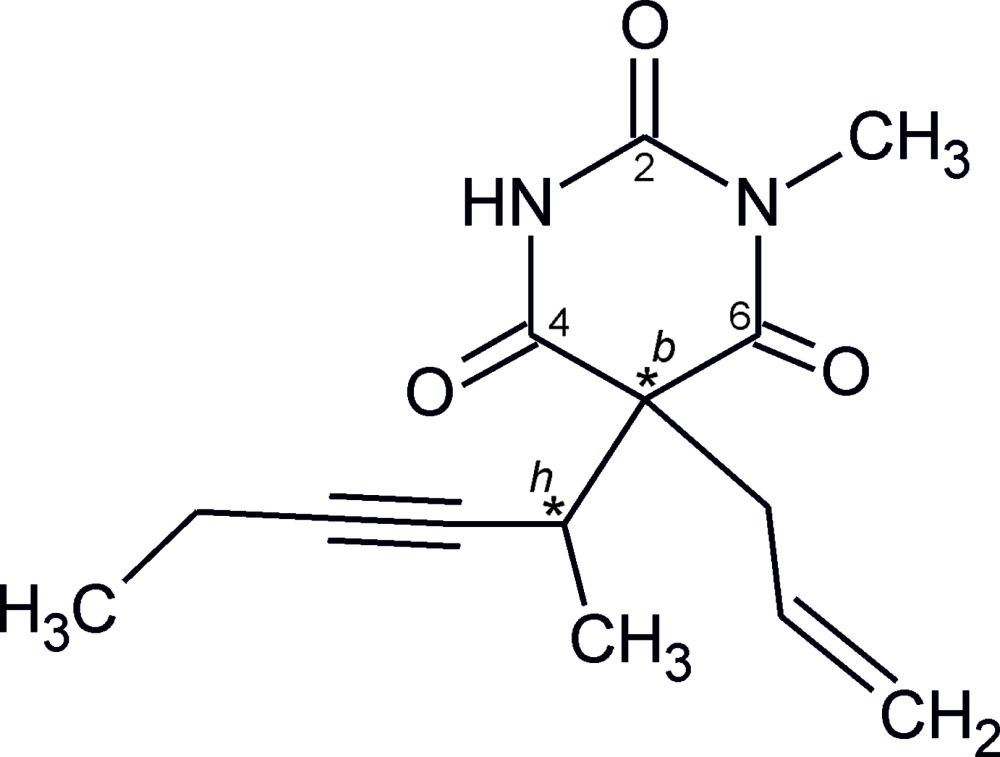


## Structural commentary   

**Figure 1:**
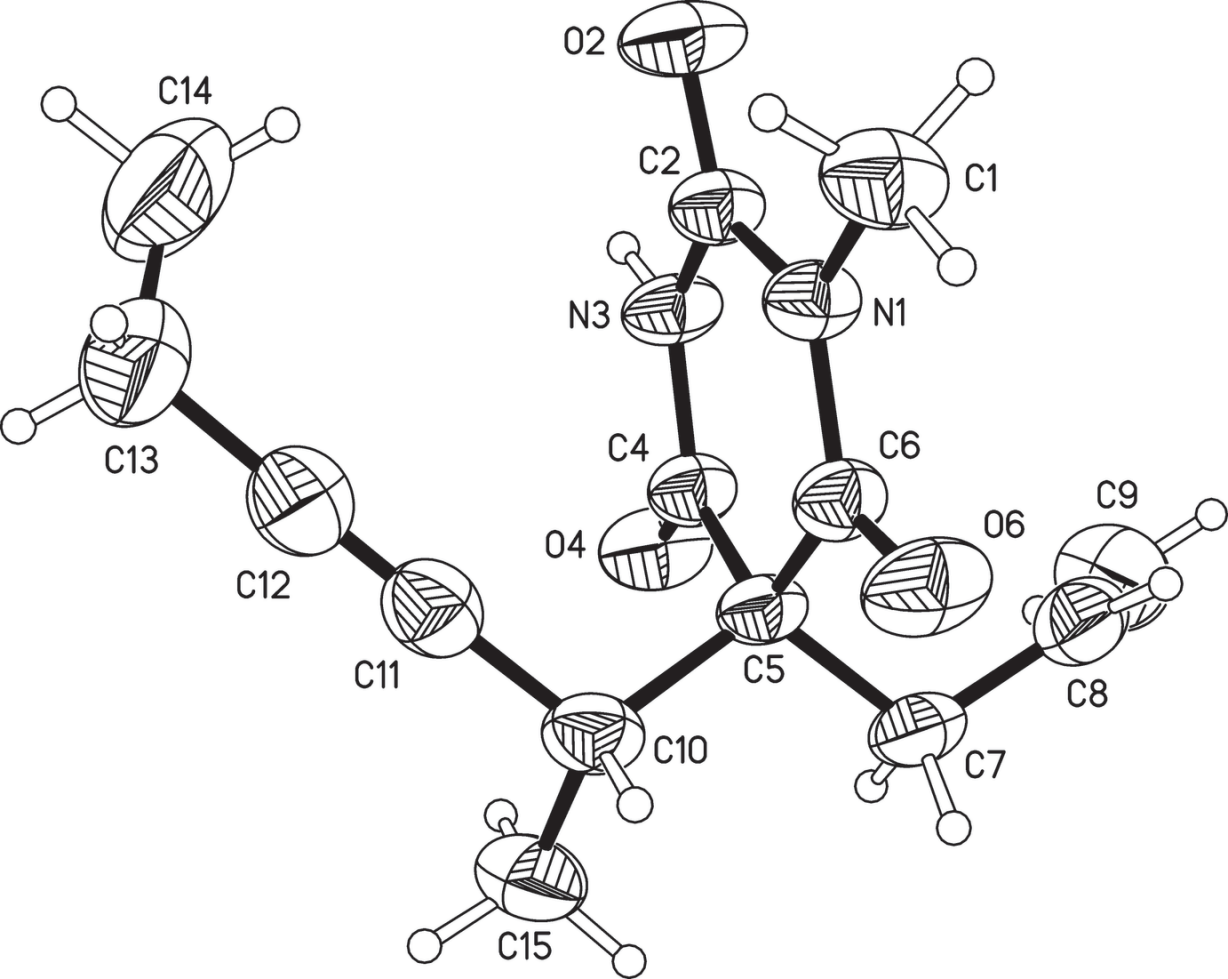
The mol­ecular structure of the title compound with displacement ellipsoids drawn at the 50% probability level; hydrogen atoms are drawn as spheres of arbitrary size.

This study confirmed the presence of the (*R_b_S_h_*)/(*S_b_R_h_*) racemate. The mol­ecule (Fig. 1 ▸) displays an approximately planar pyrimidine­trione unit in which the oxygen atoms of the C2 and C4 carbonyl groups lie at distances of −0.160 (2) and 0.156 (2) Å from the mean plane of the six-membered ring (r.m.s. deviation = 0.046 Å). The conformation of the two 5-substituents of the ring is characterized by three parameters, the torsion angles C5—C7—C8—C9 of −103.3 (2) and C10—C5—C7—C8 of −171.51 (13)° and the pseudo-torsion angle C5—C10⋯C13–C14 of 23.2 (2)°.

**Figure 2:**
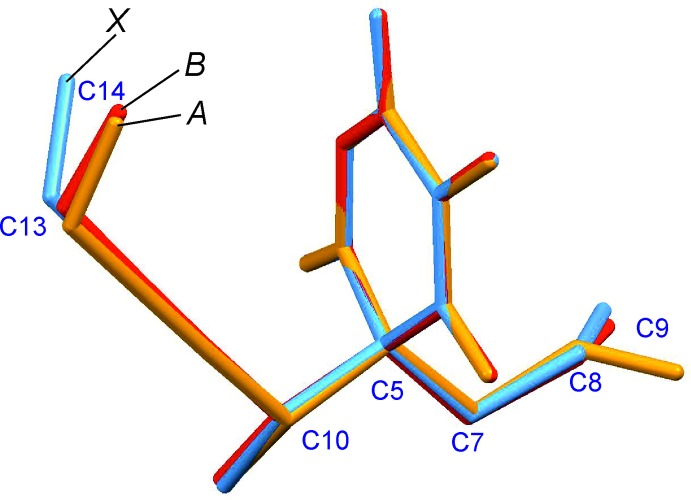
Overlay of the mol­ecule of the α-racemate (denoted *X*) with the two independent mol­ecules (*A*, *B*) of the previously reported (*S_b_R_h_*) form, generated by least-squares fits of their 1-methyl-2,4,6-pyrimidine­trione units (ten non-H atomic positions).

The previously reported (*S_b_R_h_*) form contains two independent mol­ecules (denoted *A* and *B*), which differ from the mol­ecule of the title structure in the conformation adopted by the terminal groups of both 5-substituents (Fig. 2 ▸). Specifically, in mol­ecule *A*, the torsion angle analogous to C5—C7—C8—C9 in the present α-racemate is 125.3°, and the pseudo-torsion angles analogous to C5—C10⋯C13—C14 of the title structure are −15.4° (*A*) and −26.3° (*B*).

## Supra­molecular features   

**Figure 3:**
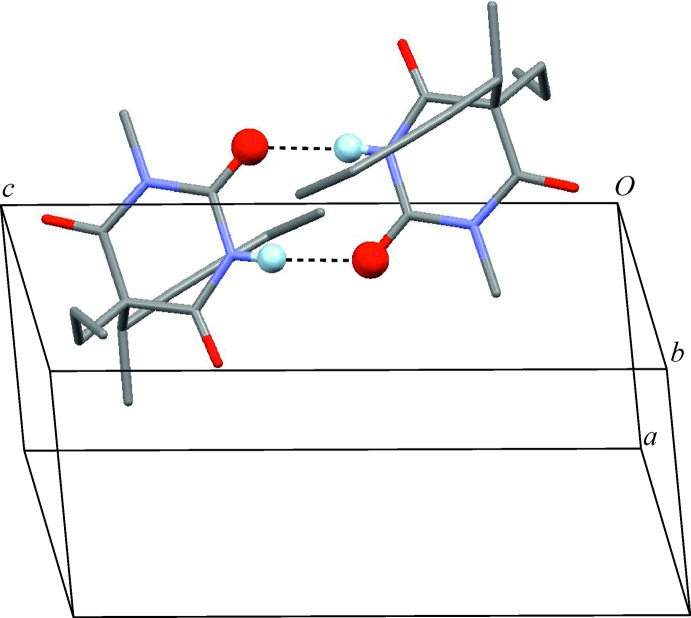
The N—H⋯O=C hydrogen-bonded inversion dimer displaying a central 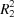
(8) ring. These inter­actions (dotted lines) involve the carbonyl group at the 2-position of the six-membered ring. O and H atoms engaged in hydrogen bonding are drawn as spheres.

Two mol­ecules are linked to one another by two mutual anti­parallel N—H⋯O=C bonds so that an inversion dimer is formed (Table 1 ▸, Fig. 3 ▸), which displays a central 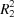
(8) ring motif (Etter *et al.*, 1990 ▸; Bernstein *et al.*, 1995 ▸). This inter­action involves the carbonyl group at the 2-position of the ring. The 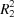
(8) ring motif is also present in the (*S_b_R_h_*) form (Brunner *et al.*, 2003 ▸) where it connects the two crystallographically independent mol­ecules. However, in this case the dimer is based on two topologically distinct N—H⋯O=C inter­actions which involve the carbonyl groups at the 4-position of the ring of mol­ecule *A* and at the 2-position of mol­ecule *B*.

## Database survey   

The Cambridge Structural Database (Groom & Allen, 2014 ▸; Version 3.35) contains 11 unique entries for derivatives of barbituric acid which are analogous to the title compound and substituted at the 1-position, but not at the 3-position of the six-membered ring. A common characteristic of these compounds is the presence of one hydrogen-bond donor group (NH) and three potential acceptor groups, *viz.* the carbonyl groups at the ring positions 2, 4 and 6. Thus, three topologically distinct hydrogen-bonding acceptor inter­actions are possible. Additionally, there is a competition between possible dimer and catemer motifs, which is similar to the competition between hydrogen-bonded dimer and catemer motifs between carboxyl groups (Beyer & Price; 2000 ▸) or carboxamide groups (Arlin *et al.*, 2010 ▸, 2011 ▸).

**Figure 4:**
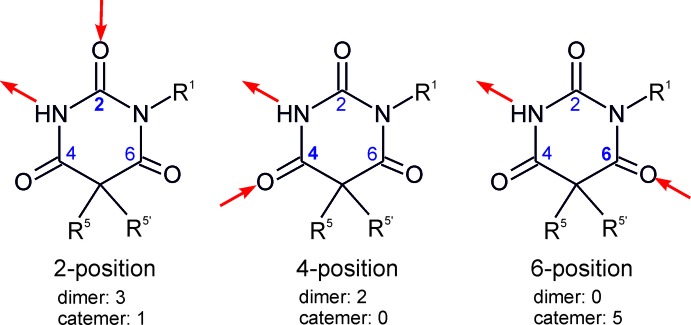
The three fundamental connection modes for the formation of N—H⋯O=C bonds in 1-substituted derivatives of barbituric acid arising from the involvement of different carbonyl groups, and the corresponding numbers of observed dimer and catemer isomers. The (*S_b_R_h_*) form of methohexithal contains a dimer with mixed N—H⋯O=C2/N—H⋯O=C4 connectivity and was therefore not included.

Closer inspection of the geometric possibilities (Fig. 4 ▸) shows that dimer formation is feasible for N—H⋯O=C2 and N—H⋯O=C4 connections only, whereas N—H⋯O=C6 should be the preferred connection mode for chain formation. Indeed, five crystal structures containing N—H⋯O=C6 chain motifs are known and their CSD refcodes are DMCYBA01 (Nichol & Clegg, 2005 ▸), DULMED (Gelbrich *et al.*, 2010 ▸), MDEBAR (Wunderlich, 1973 ▸), MIBABA (Wilhelm & Fischer, 1976 ▸), OBIPUM (Gelbrich & Griesser, 2009 ▸). So far, the crystal structure with refcode VEMQUB (Savechenkov *et al.*, 2012 ▸) is the only example in the set where another chain type, *viz.* N—H⋯O=C2, is present.

Apart from the title structure, two analogues with refcodes CXALBA (Dideberg *et al.*, 1975 ▸) and DULMAZ (Gelbrich *et al.*, 2010 ▸) also form N—H⋯O=C2 bonded dimers. The alternative N—H⋯O=C4 dimer was observed in the two structures with refcodes ALLBTC (Pyżalska *et al.*, 1980 ▸) and MEPBAB01 (Lewis *et al.*, 2005 ▸). The (*S_b_R_h_*) form of methohexital provides the only case of a dimer based on a mixed N—H⋯O=C2/N—H⋯O=C4 connectivity.

## Synthesis and crystallization   

The crystals investigated in this study were obtained at room temperature, by slow evaporation from an aqueous solution of the α-racemate of methohexital (Lilly Research Centre Ltd., Windlesham, England).

## Refinement   

Crystal data, data collection and structure refinement details are summarized in Table 2 ▸. H atoms were identified in difference maps. The H atoms of the C14 methyl group and disordered C1 methyl group [occupancy ratio 0.57 (2):0.43 (2)] were idealized and included as rigid groups allowed to rotate but not tip (C—H = 0.96 Å) and refined with *U*_iso_ set to 1.5*U*_eq_(C) of the parent carbon atom. H atoms bonded to secondary CH_2_ (C—H = 0.97 Å), tertiary CH (C—H = 0.98 Å) carbon and aromatic CH carbon atoms (C—H = 0.93 Å) were positioned geometrically and refined with *U*_iso_ set to 1.2*U*_eq_(C) of the parent carbon atom. The NH hydrogen atom was refined with a restrained distance [N—H = 0.86 (2) Å] and its *U*_iso_ parameter was freely refined.

## Supplementary Material

Crystal structure: contains datablock(s) I. DOI: 10.1107/S205698901500105X/wm5105sup1.cif

Structure factors: contains datablock(s) I. DOI: 10.1107/S205698901500105X/wm5105Isup2.hkl

Supporting information file. DOI: 10.1107/S205698901500105X/wm5105Isup3.cml

CCDC reference: 1044166

Additional supporting information:  crystallographic information; 3D view; checkCIF report

## Figures and Tables

**Table 1:** Hydrogen-bond geometry (, )

*D*H*A*	*D*H	H*A*	*D* *A*	*D*H*A*
N3H3O2^i^	0.85(2)	2.03(2)	2.8826(17)	173.2(17)

Symmetry code: (i) 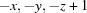.

**Table 2:** Experimental details

Crystal data	
Chemical formula	C_14_H_18_N_2_O_3_
*M*_r_	262.30
Crystal system, space group	Triclinic, *P* 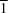
Temperature (K)	293
*a*, *b*, *c* ()	7.7502(6), 7.9792(5), 12.6881(10)
, , ()	93.713(6), 96.226(6), 113.314(7)
*V* (^3^)	711.32(10)
*Z*	2
Radiation type	Mo *K*
(mm^1^)	0.09
Crystal size (mm)	0.35 0.20 0.20

Data collection	
Diffractometer	Agilent Xcalibur (Ruby, Gemini ultra)
Absorption correction	Multi-scan (*CrysAlis PRO*; Agilent, 2012 ▸)
*T*_min_, *T*_max_	0.883, 1.000
No. of measured, independent and observed [*I* > 2(*I*)] reflections	6896, 3363, 2462
*R*_int_	0.022
(sin /)_max_ (^1^)	0.690

Refinement	
*R*[*F* ^2^ > 2(*F* ^2^)], *wR*(*F* ^2^), *S*	0.049, 0.135, 1.05
No. of reflections	3363
No. of parameters	180
H-atom treatment	H atoms treated by a mixture of independent and constrained refinement
_max_, _min_ (e ^3^)	0.22, 0.20

Computer programs: *CrysAlis PRO* (Agilent, 2012 ▸), *SHELXS97* (Sheldrick, 2008 ▸), *SHELXL2014*/6 (Sheldrick, 2015 ▸), *XP* in *SHELXTL* (Sheldrick, 2008 ▸), *Mercury* (Macrae *et al.*, 2006 ▸) and *publCIF* (Westrip, 2010 ▸).

## supplementary crystallographic information

### Crystal data

**Table:**

C_14_H_18_N_2_O_3_	*Z* = 2
*M**_r_* = 262.30	*F*(000) = 280
Triclinic, *P*1	*D*_x_ = 1.225 Mg m^−^^3^
*a* = 7.7502 (6) Å	Mo *K*α radiation, λ = 0.71073 Å
*b* = 7.9792 (5) Å	Cell parameters from 1814 reflections
*c* = 12.6881 (10) Å	θ = 4.4–28.8°
α = 93.713 (6)°	µ = 0.09 mm^−^^1^
β = 96.226 (6)°	*T* = 293 K
γ = 113.314 (7)°	Prism, colourless
*V* = 711.32 (10) Å^3^	0.35 × 0.20 × 0.20 mm

### Data collection

**Table:**

Agilent Xcalibur (Ruby, Gemini ultra) diffractometer	3363 independent reflections
Radiation source: Enhance (Mo) X-ray Source	2462 reflections with *I* > 2σ(*I*)
Graphite monochromator	*R*_int_ = 0.022
Detector resolution: 10.3575 pixels mm^-1^	θ_max_ = 29.4°, θ_min_ = 2.8°
ω scans	*h* = −9→10
Absorption correction: multi-scan (*CrysAlis PRO*; Agilent, 2012)	*k* = −11→9
*T*_min_ = 0.883, *T*_max_ = 1.000	*l* = −16→15
6896 measured reflections	

### Refinement

**Table:**

Refinement on *F*^2^	0 restraints
Least-squares matrix: full	Hydrogen site location: mixed
*R*[*F*^2^ > 2σ(*F*^2^)] = 0.049	H atoms treated by a mixture of independent and constrained refinement
*wR*(*F*^2^) = 0.135	*w* = 1/[σ^2^(*F*_o_^2^) + (0.0572*P*)^2^ + 0.1234*P*] where *P* = (*F*_o_^2^ + 2*F*_c_^2^)/3
*S* = 1.05	(Δ/σ)_max_ < 0.001
3363 reflections	Δρ_max_ = 0.22 e Å^−^^3^
180 parameters	Δρ_min_ = −0.19 e Å^−^^3^

### Special details

**Table:**

Geometry. All e.s.d.'s (except the e.s.d. in the dihedral angle between two l.s. planes) are estimated using the full covariance matrix. The cell e.s.d.'s are taken into account individually in the estimation of e.s.d.'s in distances, angles and torsion angles; correlations between e.s.d.'s in cell parameters are only used when they are defined by crystal symmetry. An approximate (isotropic) treatment of cell e.s.d.'s is used for estimating e.s.d.'s involving l.s. planes.
Refinement. The C1 methyl group is disordered over two positions.

### Fractional atomic coordinates and isotropic or equivalent isotropic displacement parameters (Å^2^)

**Table:**

	*x*	*y*	*z*	*U*_iso_*/*U*_eq_	Occ. (<1)
N1	−0.00769 (16)	−0.12155 (16)	0.75511 (9)	0.0382 (3)	
N3	0.15035 (17)	0.08665 (17)	0.63981 (10)	0.0412 (3)	
H3	0.145 (2)	0.115 (2)	0.5763 (16)	0.055 (5)*	
O2	−0.12784 (15)	−0.15309 (16)	0.58085 (9)	0.0582 (4)	
O4	0.42793 (15)	0.32706 (15)	0.69135 (9)	0.0546 (3)	
O6	0.12730 (17)	−0.09825 (17)	0.92461 (9)	0.0589 (3)	
C1	−0.1759 (2)	−0.2810 (2)	0.77286 (14)	0.0550 (4)	
H1A	−0.1624	−0.3036	0.8461	0.082*	0.43 (2)
H1B	−0.2871	−0.2566	0.7570	0.082*	0.43 (2)
H1C	−0.1881	−0.3870	0.7272	0.082*	0.43 (2)
H1D	−0.2627	−0.3279	0.7074	0.082*	0.57 (2)
H1E	−0.1380	−0.3749	0.7965	0.082*	0.57 (2)
H1F	−0.2370	−0.2445	0.8263	0.082*	0.57 (2)
C2	−0.0015 (2)	−0.06791 (19)	0.65385 (11)	0.0393 (3)	
C4	0.30668 (19)	0.18923 (19)	0.71322 (11)	0.0376 (3)	
C5	0.32002 (19)	0.11774 (19)	0.82021 (11)	0.0368 (3)	
C6	0.1393 (2)	−0.04093 (19)	0.83867 (11)	0.0383 (3)	
C7	0.3687 (2)	0.2764 (2)	0.90965 (12)	0.0460 (4)	
H7A	0.3993	0.2375	0.9775	0.055*	
H7B	0.4802	0.3803	0.8970	0.055*	
C8	0.2092 (2)	0.3359 (2)	0.91640 (13)	0.0527 (4)	
H8	0.1068	0.2623	0.9479	0.063*	
C9	0.2045 (3)	0.4828 (3)	0.88144 (18)	0.0759 (6)	
H9A	0.3047	0.5594	0.8496	0.091*	
H9B	0.1010	0.5118	0.8882	0.091*	
C10	0.4766 (2)	0.0383 (2)	0.82568 (12)	0.0463 (4)	
H10	0.4680	−0.0253	0.8897	0.057 (5)*	
C11	0.4322 (2)	−0.1006 (2)	0.73331 (14)	0.0510 (4)	
C12	0.3973 (3)	−0.2041 (3)	0.65519 (17)	0.0620 (5)	
C13	0.3521 (4)	−0.3299 (3)	0.55608 (19)	0.0882 (7)	
H13A	0.4699	−0.3204	0.5319	0.106*	
H13B	0.2858	−0.4551	0.5716	0.106*	
C14	0.2356 (5)	−0.2930 (4)	0.4700 (2)	0.1143 (10)	
H14A	0.2091	−0.3809	0.4086	0.171*	
H14B	0.3027	−0.1714	0.4518	0.171*	
H14C	0.1185	−0.3025	0.4931	0.171*	
C15	0.6801 (2)	0.1819 (3)	0.83485 (16)	0.0640 (5)	
H15A	0.7656	0.1217	0.8353	0.096*	
H15B	0.7106	0.2628	0.8999	0.096*	
H15C	0.6922	0.2517	0.7751	0.096*	

### Atomic displacement parameters (Å^2^)

**Table:**

	*U*^11^	*U*^22^	*U*^33^	*U*^12^	*U*^13^	*U*^23^
N1	0.0343 (6)	0.0402 (6)	0.0336 (6)	0.0085 (5)	0.0022 (5)	0.0091 (5)
N3	0.0409 (7)	0.0458 (7)	0.0270 (6)	0.0083 (5)	−0.0027 (5)	0.0108 (5)
O2	0.0463 (6)	0.0661 (7)	0.0372 (6)	−0.0001 (5)	−0.0087 (5)	0.0094 (5)
O4	0.0494 (6)	0.0501 (6)	0.0469 (7)	0.0019 (5)	0.0011 (5)	0.0168 (5)
O6	0.0592 (7)	0.0696 (8)	0.0345 (6)	0.0109 (6)	0.0028 (5)	0.0210 (5)
C1	0.0428 (8)	0.0541 (9)	0.0541 (10)	0.0037 (7)	0.0054 (7)	0.0182 (8)
C2	0.0366 (7)	0.0430 (8)	0.0328 (8)	0.0117 (6)	−0.0010 (6)	0.0069 (6)
C4	0.0357 (7)	0.0389 (7)	0.0326 (7)	0.0100 (6)	0.0014 (6)	0.0072 (6)
C5	0.0351 (7)	0.0424 (7)	0.0276 (7)	0.0115 (6)	−0.0017 (5)	0.0054 (5)
C6	0.0396 (7)	0.0429 (8)	0.0302 (7)	0.0143 (6)	0.0026 (6)	0.0081 (6)
C7	0.0433 (8)	0.0508 (9)	0.0329 (8)	0.0110 (7)	−0.0046 (6)	−0.0011 (6)
C8	0.0504 (9)	0.0550 (10)	0.0433 (9)	0.0138 (8)	0.0031 (7)	−0.0034 (7)
C9	0.0711 (13)	0.0635 (12)	0.0904 (16)	0.0273 (10)	0.0032 (11)	0.0041 (11)
C10	0.0434 (8)	0.0571 (9)	0.0387 (8)	0.0215 (7)	−0.0007 (6)	0.0112 (7)
C11	0.0491 (9)	0.0562 (10)	0.0533 (10)	0.0268 (8)	0.0064 (8)	0.0122 (8)
C12	0.0633 (11)	0.0639 (11)	0.0663 (12)	0.0352 (9)	0.0044 (9)	0.0051 (9)
C13	0.1023 (18)	0.0897 (16)	0.0816 (16)	0.0565 (14)	−0.0012 (14)	−0.0187 (13)
C14	0.146 (3)	0.130 (2)	0.0667 (16)	0.064 (2)	0.0027 (17)	−0.0222 (15)
C15	0.0396 (9)	0.0777 (12)	0.0671 (12)	0.0194 (9)	−0.0034 (8)	0.0029 (10)

### Geometric parameters (Å, º)

**Table:**

N1—C2	1.3800 (18)	C7—H7A	0.9700
N1—C6	1.3804 (17)	C7—H7B	0.9700
N1—C1	1.4687 (18)	C8—C9	1.292 (3)
N3—C2	1.3648 (18)	C8—H8	0.9300
N3—C4	1.3701 (17)	C9—H9A	0.9300
N3—H3	0.85 (2)	C9—H9B	0.9300
O2—C2	1.2140 (16)	C10—C11	1.471 (2)
O4—C4	1.2032 (17)	C10—C15	1.526 (2)
O6—C6	1.2083 (17)	C10—H10	0.9800
C1—H1A	0.9600	C11—C12	1.182 (2)
C1—H1B	0.9600	C12—C13	1.474 (3)
C1—H1C	0.9600	C13—C14	1.460 (3)
C1—H1D	0.9600	C13—H13A	0.9700
C1—H1E	0.9600	C13—H13B	0.9700
C1—H1F	0.9600	C14—H14A	0.9600
C4—C5	1.5151 (18)	C14—H14B	0.9600
C5—C6	1.5248 (19)	C14—H14C	0.9600
C5—C7	1.541 (2)	C15—H15A	0.9600
C5—C10	1.574 (2)	C15—H15B	0.9600
C7—C8	1.498 (2)	C15—H15C	0.9600
			
C2—N1—C6	123.81 (12)	O6—C6—C5	120.29 (12)
C2—N1—C1	117.84 (12)	N1—C6—C5	119.14 (12)
C6—N1—C1	118.24 (12)	C8—C7—C5	112.61 (12)
C2—N3—C4	127.42 (12)	C8—C7—H7A	109.1
C2—N3—H3	113.2 (12)	C5—C7—H7A	109.1
C4—N3—H3	119.3 (12)	C8—C7—H7B	109.1
N1—C1—H1A	109.5	C5—C7—H7B	109.1
N1—C1—H1B	109.5	H7A—C7—H7B	107.8
H1A—C1—H1B	109.5	C9—C8—C7	124.36 (18)
N1—C1—H1C	109.5	C9—C8—H8	117.8
H1A—C1—H1C	109.5	C7—C8—H8	117.8
H1B—C1—H1C	109.5	C8—C9—H9A	120.0
N1—C1—H1D	109.5	C8—C9—H9B	120.0
H1A—C1—H1D	141.1	H9A—C9—H9B	120.0
H1B—C1—H1D	56.3	C11—C10—C15	110.95 (15)
H1C—C1—H1D	56.3	C11—C10—C5	109.25 (12)
N1—C1—H1E	109.5	C15—C10—C5	114.96 (14)
H1A—C1—H1E	56.3	C11—C10—H10	107.1
H1B—C1—H1E	141.1	C15—C10—H10	107.1
H1C—C1—H1E	56.3	C5—C10—H10	107.1
H1D—C1—H1E	109.5	C12—C11—C10	176.00 (18)
N1—C1—H1F	109.5	C11—C12—C13	178.4 (2)
H1A—C1—H1F	56.3	C14—C13—C12	113.7 (2)
H1B—C1—H1F	56.3	C14—C13—H13A	108.8
H1C—C1—H1F	141.1	C12—C13—H13A	108.8
H1D—C1—H1F	109.5	C14—C13—H13B	108.8
H1E—C1—H1F	109.5	C12—C13—H13B	108.8
O2—C2—N3	121.50 (13)	H13A—C13—H13B	107.7
O2—C2—N1	121.22 (13)	C13—C14—H14A	109.5
N3—C2—N1	117.27 (12)	C13—C14—H14B	109.5
O4—C4—N3	120.57 (12)	H14A—C14—H14B	109.5
O4—C4—C5	122.59 (12)	C13—C14—H14C	109.5
N3—C4—C5	116.82 (12)	H14A—C14—H14C	109.5
C4—C5—C6	114.41 (11)	H14B—C14—H14C	109.5
C4—C5—C7	108.82 (12)	C10—C15—H15A	109.5
C6—C5—C7	108.29 (12)	C10—C15—H15B	109.5
C4—C5—C10	108.59 (12)	H15A—C15—H15B	109.5
C6—C5—C10	105.19 (11)	C10—C15—H15C	109.5
C7—C5—C10	111.55 (11)	H15A—C15—H15C	109.5
O6—C6—N1	120.54 (13)	H15B—C15—H15C	109.5
			
C10—C5—C7—C8	−171.51 (13)	C5—C7—C8—C9	−103.3 (2)

### Hydrogen-bond geometry (Å, º)

**Table:**

*D*—H···*A*	*D*—H	H···*A*	*D*···*A*	*D*—H···*A*
N3—H3···O2^i^	0.85 (2)	2.03 (2)	2.8826 (17)	173.2 (17)

Symmetry code: (i) −*x*, −*y*, −*z*+1.
